# Orthorexia Nervosa and Disordered Eating Attitudes, Self-Esteem and Physical Activity among Young Adults

**DOI:** 10.3390/nu14061289

**Published:** 2022-03-18

**Authors:** Anna Brytek-Matera, Susanna Pardini, Joanna Szubert, Caterina Novara

**Affiliations:** 1 Eating Behavior Laboratory (EAT Lab), Institute of Psychology, University of Wrocław, 50-527 Wrocław, Poland; joanna.szubert@uwr.edu.pl; 2 Department of General Psychology, University of Padova, 35131 Padova, Italy; susanna.pardini@phd.unipd.it

**Keywords:** orthorexia nervosa, dieting, self-esteem, physical activity

## Abstract

A relation between Orthorexia Nervosa (ON) and increased frequency of physical activity has been put in evidence by recent studies. It is well known that intense physical exercises are typically related to eating disorders, but its relationship with ON is still a subject of debate. Other transdiagnostic features could be necessary to conceptualize and understand ON; in this way, low self-esteem is related to eating behavior but is not still extensively investigated in ON, and, to date, data are so heterogeneous that they do not allow us to understand if this is a psychological feature somehow associated with ON. The current study aimed to assess whether disordered eating attitudes, self-esteem, and physical activity are associated with ON in young adults from Poland and Italy. Moreover, we investigated the differences by comparing lower and higher ON levels related to disordered eating attitudes, self-esteem, and physical activity. Our results indicated that a great concern about dieting significantly predicted problems associated with healthy eating, knowledge about healthy eating, and feeling positive about healthy eating. In addition, young adults with a high level of ON demonstrated higher levels of disordered eating attitudes and vigorous-intensity physical activity than young adults with a low level of ON. Future studies are needed to assess the direct effect of physical activity and self-esteem on ON.

## 1. Introduction

The first publication describing orthorexia nervosa (ON) appeared in the late 1990s. Bratman and Knight [1] defined ON as “a fixation on eating healthy food” (p. 9) with the aim of achieving healthiness (and/or avoiding illness). ON is marked by a rigid avoidance of foods considered by the individual to be impure or unhealthy (e.g., saturated fats), a constant preoccupation with dietary practices (which leads to the elimination of an entire food group), very inflexible dietary rules with violations resulting in excessive emotional distress, health problems, and psychological disturbances [2,3]. Although ON has been studied successively in the last two decades and is achieving expanding scientific attention, it has up to now not been identified neither by the Diagnostic and Statistical Manual of Mental Disorders [4] nor by the International Classification of Diseases [5]. This may be elucidated by the current state of research provided inconsistent results (related to sex, age, body mass index, and ON prevalence [6,7]) but also by the still-evolving debate about the conceptualization of ON (definition and diagnostic criteria) and its classification among other mental disorders (a distinct disorder, a variant of eating disorder or a variant of obsessive-compulsive disorder).

Previous studies have found that ON is related to symptoms and concerns characteristic of eating disorders [8,9,10,11,12,13,14]. A recent study [8] has shown that eating disorder pathology was found to be related to ON among Polish and Spanish university students, suggesting that ON should be considered as disordered eating behavior closely connected with eating disorders (EDs). These results are in line with other studies showing that greater ON symptomatology is associated with greater disordered eating among adults from the general population [11,12,15,16,17]. It is worth pointing out that the recent systematic review and meta-analysis [18] on relationship between ON and disordered eating, and obsessive–compulsive (OCD) symptoms have demonstrated a moderate association between ON and EDs symptoms and a small association between ON and OCD symptoms. In addition, ON has been found to be more associated with EDs compared to OCD.

Previous studies have revealed that increased ON symptomatology corresponds to increased exercise frequency [19] and sport participation [20]. Regarding quantitative correlational research, three studies found significant positive correlations between ON symptomatology and frequency of physical activity, as measured by exercise sessions per week among fitness center members [21] and by sport activity among university students [19,22]. The previous research has shown that greater ON symptomatology was associated with more time spent on both aerobic and strength-training exercises as well as with greater levels of exercise addiction and compulsion [23], which is consistent with the results of one other study finding a significant relationship between ON and exercise addiction in a sample of fitness center members [21].

Low self-esteem has been found to play a major role in the development of eating disorders. The recent meta-analysis [24] has shown a negative effect of self-esteem on eating disorders. Although low self-esteem acts as a universal risk factor for different eating disorders [24] the relationship between self-esteem and ON is not so obvious. Some studies [25,26] have found no relationship between ON and self-esteem. On the other hand, other research [27] has demonstrated a negative correlation. There are indications suggesting a relationship between self-esteem and ON. McGregor [28] in her clinical practice observed orthorexic behavior motivated by the desire to develop one’s potential, which increases self-esteem. According to McGregor [28], orthorexia should be better described as an obsessive self-development rather than an obsessive care for a healthy diet. Greville-Harris, Smithson, and Karl [29] conducted a qualitative study analyzing the statements of people with ON characteristics on social media. The respondents admitted that they have a sense of superiority and identify themselves as being better and healthier than others. Strict adherence to self-appointed eating practices increased self-esteem and led to a feeling of superiority over others. This is in line with the social comparison theory. People who have low self-esteem often engage in such behaviors, and their satisfaction may increase as a result [30].

The research conducted so far on factors related to ON is insufficient. A previous study [24] demonstrated that higher overweight preoccupation, higher appearance orientation and the presence of an eating disorder history (the strongest predictor) were predictors of ON among Australian university students. Another study [3] showed that pathological eating, eating style, Mediterranean diet, compulsive symptoms, and subjective social status predicted ON among German young adults, whereas weight control, emotion regulation, low sensorial appeal, and younger age predicted ON among Spanish university students [31]. The objective of the present study was to assess whether disordered eating attitudes, self-esteem, and physical activity are associated with ON in young adults (from Poland and Italy). In the present study, we also investigated ON level (low versus high) with disordered eating attitudes, self-esteem, and physical activity.

## 2. Methods

### 2.1. Participants and Procedure

The study group consisted of 243 (43.9%) Polish and 311 (56.1%) Italian university students respectively recruited from different universities located in Poland (e.g., Silesian region, Upper Silesia region) and Italy (e.g., northern Italy).

The current study is a part of a project about maladaptive eating in adulthood from a cross-cultural perspective (MEALS study). The procedure of the study has been described in detail in the previous work [31]. To sum up, the administration session took place in one session based on a series of self-report questionnaires that participants had to fill out via Google Forms. Protocols with at least 10% of the answers omitted have been excluded.

Ethical approval for the MEALS study was obtained from Polish and Italian research ethics committees (No. WKEB59/05/2019 and No. 3067-14 June 2019). The study was performed in accordance with the ethical standards as laid down in the 1964 Declaration of Helsinki and its later amendments or comparable ethical standards.

### 2.2. Measures

#### 2.2.1. Demographic Schedule

The demographics data (e.g., age, sex, Body Mass Index), together with information on diet type, omission of food, and presence or absence of medical or psychological diagnosis were collected in the present study. The presence of medical and psychological diagnoses has been considered as an exclusion criterion.

#### 2.2.2. The Eating Habits Questionnaire (EHQ): Assessment of ON

The EHQ [32] is a 21-item self-report questionnaire on a four-point Likert scale, evaluating cognitions, feelings and behaviors related to ON. Specifically, items are grouped into three subscales: “Knowledge” (referring to knowledge and beliefs related to healthy eating), “Feelings” (feelings, emotions, and sensations associated with following a healthy diet), and “Problems” (difficulties and problems related to the healthy diet pursued). The EHQ [32] demonstrated satisfactory internal consistency (0.82 < Cronbach’s α < 0.90) and test–retest reliability (0.72 < r < 0.81).

In the present study, we use the Polish version [33] and the Italian version [34] of the EHQ. Reliability analysis for Polish versions of the EHQ across the whole questionnaire showed strong internal consistency (Cronbach’s α EHQ-PL = 0.88); moreover, considering the Italian version, both exploratory and confirmatory factor analyses have shown that the EHQ-21 was a reliable measure (fit indices: Problems (CFI = 0.99; NNFI = 0.99; RMSEA = 0.06); Knowledge (CF = 0.99; NNFI = 0.97; RMSEA = 0.12); Feelings (CFI = 0.99; NNFI = 0.97; RMSEA = 0.08); Total (CFI = 0.99; NNFI = 0.99; RMSEA = 0.05), and also the between scales’ intercorrelations were adequate (Problems-Knowledge = 0.71; Problems-Feelings = 0.86; Knowledge-Feelings = 0.64)).

This study demonstrated Polish and Italian samples’ adequate reliability (for total and subscales scores, Cronbach’s α ranging from 0.74 and 0.90).

#### 2.2.3. The Eating Attitudes Test (EAT-26): Assessment of Disordered Eating Attitudes

The EAT-26 [35] is a widely used 26-item self-report screening questionnaire investigating the presence of “eating disorder risk” based on attitudes related to disordered eating. The EAT-26 is composed of three subscales: dieting (restricting intake of high caloric foods and preoccupation with body image/shape), bulimia and food preoccupation (thoughts regarding food, binging and self-induced vomiting), and oral control (self-control of eating and perceived pressure from others to gain weight). Higher scores indicate a greater risk of an eating disorder and a total score of 20 or above is considered to be in the clinical range. In the present study, we use the Polish version [36] and the Italian version [37] of the EAT-26. Reliability analysis for two versions of the EAT-26 across the whole questionnaire shows strong internal consistency (Cronbach’s α EAT-26-PL = 0.80; Cronbach’s α EAT26-IT = 0.86). This study demonstrated Polish and Italian samples’ adequate reliability (for total and subscales scores, Cronbach’s α ranging from 0.65 and 0.87).

#### 2.2.4. The Rosenberg Self-Esteem Scale (RSES): Assessment of Self-Esteem

The RSES [38] is a widely used 10-item self-report questionnaire measuring features related to self-esteem. In the present study we use the Polish version [39] and the Italian version [40] of the RSES. Reliability analysis for two versions of the RSESE shows strong internal consistency (Cronbach’s α RSES-PL = 0.82; Cronbach’s α RSES-IT = 0.84). This study demonstrated Polish and Italian samples’ adequate reliability (Cronbach’s α = 0.88).

#### 2.2.5. The International Physical Activity Questionnaire (IPAQ): Assessment of Physical Activity

The short form of the IPAQ is a widely used 7-item standardized self-rating questionnaire assessing physical activity (walking, moderate-intensity activity and vigorous-intensity activity) based on recall over the previous week. The intensity of physical activity is classified according to Metabolic Equivalent of Task (MET) cut-offs of 3.3 for walking, MET 4 for moderate-intensity physical activity, and MET 8 for vigorous-intensity physical activity [41]. The total physical activity score is obtained by summing up walking, moderate and vigorous MET-minutes/week scores.

In the present study, we used the adaptation of IPAQ-short into Polish [42] and Italian [43] that followed the IPAQ committee guidelines [41].

## 3. Results

### 3.1. Demographic Features of the University Students’ Samples

The main characteristics of young adults in Poland and in Italy are described in Table 1.

Regarding other significant features such as smoking habit, use of drugs (currently and in the past) or alcohol, allergies, food intolerance, learning disabilities, body image satisfaction, habits of weighing every day, and lose weight intentionally, no differences were put in evidence between the two groups. (In the Polish group: 33 (13.6%) had a food intolerance, 144 (59.3%) students affirmed to be satisfied with their body image, 17 (7%) weighed every day, 180 (74.1%) assumed to never intentionally lose weight, and 36 (87.2%) had a fasting modality. In the Italian group: 51 (16.4%) had a food intolerance, 164 (52.7%) students affirmed to be satisfied with their body image. 33 (10.6%) weighted every day. 231 (74.3%) never assumed to lose weight intentionally.) Regarding other significant features in the total sample, 84 (15.2%) had a food intolerance, 308 (55.6%) students affirmed to be satisfied with their body image, 50 (9%) weighed every day, and 411 (74.2%) assumed to never intentionally lose weight.

### 3.2. Invariance’s Measurement Across Groups

Considering that our sample was composed of individuals from different countries, the invariance measurement across groups was investigated. Based on the EHQ, the invariance models could be considered adequate. Indeed, although the last analysis concerning the mean invariance was not satisfied, the estimation could be regarded as acceptable (Table 2).

### 3.3. Constructs Associated with ON

A linear regression analysis based on the stepwise method was conducted for the entire group to investigate whether the EAT-26 subscales scores, the RSES total score and different intensity levels of physical activity are associated with ON considering, one by one, the EHQ total and subscales as dependent variables.

The EAT-26 Dieting subscale score is associated with the EHQ total score (R^2^ = 0.336; F(1116) = 58.127; *p* < 0.001). A significant association is also due to the IPAQ-Moderate physical activity combining with the EAT-26 Dieting subscale (R^2^ modified = 0.023; F(2116) = 31.871; *p* < 0.001).

The EHQ Problems subscale is associated with the EAT-26 Dieting subscale (R^2^ = 0.304; F(1116) = 50.245; *p* < 0.001). A significant association is also due to the EAT-26 Bulimia and Food Preoccupation subscale (R^2^ modified = 0.076; F(2116) = 34.929; *p* < 0.001), and to the IPAQ-Moderate physical activity score (R^2^ modified = 0.057; F(3116) = 29.254; *p* < 0.001).

The EHQ Knowledge is associated with the EAT-26 Dieting (R^2^ = 0.165; F(1116) = 22.770; *p* < 0.001), with a further association with the RSES total score (R^2^ modified = 0.038; F(2116) = 14.532; *p* < 0.001), and the EAT-26 Oral Control score (R^2^ = 0.033; F(3116) = 11.622; *p* < 0.001).

Finally, also for the EHQ Feelings, the better association is with the EAT-26 Dieting (R^2^ = 0.169; F(1116) = 23.385; *p* < 0.001). (Table 3).

### 3.4. Comparison of High Versus Low Level of ON with Disordered Eating Attitudes, Self-Esteem and Physical Activity

Two groups of individuals (named High-EHQ and Low-EHQ, respectively) were set up. Specifically, the EHQ cut-off scores were derived by obtaining a score equal to or greater than the 90 percentiles (Polish group’s raw score ≥ 47; Italian group’s raw score ≥ 44), while the Low-EHQ individuals were casually extracted from the group with a score less than the 50 percentile (Polish group’s raw score ≤ 33; Italian group’s raw score ≤ 31).

Multivariate ANOVA has been performed considering the “Low/High EHQ” (Table 4) and, as independent variable, the EAT-26 total and subscales, the RSES total, and the IPAQ subscales scores have been considered as dependent variables.

Significant differences emerged between the “High/Low EHQ” in the EAT-26 total and subscales, the IPAQ total and “Vigorous” subscale (10.08 < F< 56.32; *p* < 0.01; 0.07 < partial η^2^ < 0.30); otherwise, significant differences did not emerge for the RSES (F = 0.38; *p* > 0.05), the IPAQ “Walking” (F = 1.68; *p* > 0.05), and “Moderate” subscales (F = 1.87; *p* > 0.05).

## 4. Discussion

The objective of the present study was to evaluate whether disordered eating attitudes, self-esteem, and physical activity are associated with ON in young adults from Poland and Italy. Our findings showed that a high level of concern about dieting is related to problems associated with healthy eating, knowledge about healthy eating, and feeling positive about healthy eating (EHQ subscales), which indicate that restricting intake of high caloric foods and preoccupation with body image/shape is linked with ON. Previous studies demonstrated that ON is associated with disordered eating attitudes [8,9,10,11,13,14,25].

In addition, thoughts about food and self-induced vomiting together with moderate-intensity physical activity were linked with problems associated with healthy eating among young adults. The results concerning moderate-intensity physical activity was unexpected. According to widely known the World Health Organization recommendations, adults aged 18–64 should engage in at least 150–300 min of moderate-intensity aerobic physical activity or at least 75–150 min of vigorous-intensity aerobic physical activity; or an equivalent combination of moderate- and vigorous-intensity activity throughout the week. In addition, WHO recommends adults to do more than the endorsed levels of moderate- to vigorous-intensity physical activity to help decrease the damaging effects of high levels of sedentary behavior on health. The assessment of physical activity is complex to get an accurate estimation and two categories of methods (self-reported questionnaires and objective method-calorimeter, accelerometer, and pedometer) are widely used to determine physical activity levels [44]. It is worth noting that in the present study, young adults were asked to complete the short self-administered version of the IPAQ. A previous study reported that the short version of the IPAQ does not appear to be a good indicator of individual physical activity behavior but is better suited for larger population-based samples [45]. In addition, previous findings have shown that the IPAQ overestimates moderate and vigorous activity in the adult population [46,47,48] or underestimates the actual levels of physical activity [49,50]. We can suppose that young adults using self-report measures could misestimate moderate-intensity physical activity, as it has been shown in other studies demonstrating that younger adults underestimated the moderate and vigorous-intensity of physical activity more than middle-aged adults [51]. Nevertheless, this hypothesis should be verified using objective methods (e.g., accelerometers).

High self-esteem and high levels of self-control of eating and the perceived pressure from others to gain weight were associated with knowledge about healthy eating among young adults. We can assume that knowledge about healthy eating may be linked with eating competence. Young adults may believe they are able to make healthy food choices to provide for their health. It can be supposed that striving to adherence and maintaining self-determined healthy diet recommendations may result in high self-esteem. Food regime may justify the nutritional practices resulting from self-discipline, perfectionism, and individual responsibility, and may create a false sense of superiority over others [1]. Our findings also correspond to the findings of the qualitative study of Greville-Harris, Smithson, and Karl [28], who demonstrated that using dietary restrictions and making public the diet, could improve self-esteem. There is worth pointing out that lower self-esteem was found to be a predictor of ON in adults performing regular fitness activity [26], and some previous research [24,25] revealed no association between self-esteem and ON. Another study has found that intense nutritional education has been found to be related to ON in patients with type 2 diabetes mellitus [52].

In addition, the present results revealed that young adults with a high level of ON demonstrated higher levels of disordered eating attitudes and vigorous-intensity physical activity than those with low level of ON. It is widely known that physical activity in higher intensity results in longer-lasting neurobiological changes, and is positively associated with cognitive and mental health [53]. The benefits of physical activity on physical and mental health are significant. Nevertheless, some studies found that excess physical activity may affect health-compromising eating behaviors [54] and people who are physically active have been found to demonstrate inappropriate eating behaviors (food restriction, self-induced vomiting and use of weight-loss drugs) [55]. ON could result from a desire to achieve optimum health; therefore, young adults with high level of ON may engage in vigorous-intensity physical activity due to its health benefits.

The female gender is more representative in both our samples. The previous systematic review and meta-analysis have demonstrated that women were more likely to report pathologically healthful eating than men [56]. Nevertheless, there is a lack of well-established gender differences in the prevalence of ON opposite to findings showing that EDs are more common in women than in men. Therefore, it is relevant to consider gender when interpreting the results.

Our study provided information on the predictors of ON, but it was not without its limitations. First, the cross-sectional nature of the study does not allow any conclusions in terms of causality for ON. Second, physical activity was estimated from the self-reported method. Thus, future research would benefit from using the objective methods to evaluate physical activity (e.g., accelerometer, pedometer).

Both Poland and Italy are classified as individualistic cultures based on Hofstede’s cultural dimensions theory (Poland with a score of 60 and Italy with a score of 76 [57]. In addition, the effect of globalization on eating behaviors [58] and on the increased health burden associated with eating disorders pathology [59] seems to be similar in these Western countries, especially among young people.

## 5. Conclusions

Our findings demonstrated that disordered eating attitudes and moderate-intensity physical activity were associated with ON among young adults from Poland and Italy. In particular, we found that: (1) dieting, bulimia, and food preoccupation and a moderate-intensity physical activity were linked with problems associated with healthy eating; (2) dieting, self-esteem, and oral control were linked with knowledge about healthy eating; and (3) dieting was linked with feeling positive about healthy eating.

There are indications that focusing on healthy eating, as well as regular and adequate physical activity is socially acceptable and has a positive impact on health. However, abnormally high levels of physical activity may have a negative impact on health, especially among women with an eating disorder [59].

Future studies are needed, especially to assess the direct effect of physical activity and self-esteem on ON.

## Figures and Tables

**Table 1:** Socio-demographic features among young adults.

	Polish Young Adults (*N* = 243)	Italian Young Adults (*N* = 311)	Total Sample (*N* = 554)	F ^(a)^ or Chi-Squared ^(b)^	*p*	Partial η2
	M (SD) or *N* (%)					
Age	22.23 (2.30)	21.95 (2.10)	22.07 (2.19)	2.21 ^(a)^	0.14	0.004
Body Mass Index	22.65 (4.36)	21.44 (3.07)	21.97 (3.73).	14.47 ^(a)^	<0.001	0.03
Gender (% female)	203 (83.5%)	248 (79.7%)	451 (81.4)	1.30 ^(b)^	0.27	0.05
Marital status (% single)	228 (93.8%)	280 (90%)	508 (91.7)	2.58 ^(b)^	0.12	0.07
Chronic disease	43 (17.7%)	24 (7.7%)	67 (12.1)	12.78 ^(b)^	0.001	0.15
Anxiety disorder	4 (1.6%)	13 (4.2%)	17 (3.1)	2.95 ^(b)^	0.13	0.073
Avoidance of food	89 (36.6%)	91 (29.3%)	180 (32.5)	3.37 ^(b)^	0.07	0.08
Using laxatives	9 (3.7%)	5 (1.6%)	14 (2.5)	2.43 ^(b)^	0.17	0.07
Vomiting	7 (2.9%)	7 (2.3%)	14 (2.5)	0.22 ^(b)^	0.79	0.02
Omnivore diet	163 (67.4%)	290 (93.2%)	453 (81.8%)	75.29 ^(b)^	<0.001	0.37
Vegetarian diet	16 (6.6%)	15 (4.8%)	31 (5.6%)			

Note*:* M = Mean; SD = Standard Deviation, ^(a)^ = Fisher’s F; ^(b)^ = Chi-Squared.

**Table 2:** DWLS estimation method.

Model	χ2	df	χ2/df	RMSEA	90% RMSEA	SRMR	CFI	Comparison	Delta χ2	Delta df	*p*-Value	Delta CFI	Decision
CI	930.375	372	2.501	0.071	(0.066–0.077)	0.096	0.979						
LI	1060.246	390	2.719	0.076	(0.071–0.082)	0.103	0.975	Model CI vs. LI	129.871	18	0.000	−0.004	Accepted
TI	1145.522	429	2.670	0.075	(0.070–0.081)	0.098	0.973	Model TI vs. LI	85.276	39	0.000	−0.002	Accepted
FVI	1176.027	432	2.722	0.076	(0.071–0.082)	0.099	0.972	Model FVI vs. TI	30.505	3	0.000	−0.001	Accepted
FCI	1236.657	435	2.843	0.079	(0.074–0.084)	0.101	0.970	Model FCI vs. FVI	60.630	3	0.000	−0.002	Accepted
FMI	1443.375	438	3.295	0.088	(0.083–0.093)	0.102	0.962	Model FMI vs. FCI	206.718	3	0.000	−0.008	Reject

Note: CI = Full Configural Invariance; LI = Full Factor Loadings Invariance; TI = Full Thresholds Invariance; FVI = Full Factor Variance Invariance; FCI = Full Factor Covariance Invariance; FMI = Full Factor Mean Invariance; RMSEA = Root Mean Square Error of Approximation; SRMR= Standardized Root Mean Square Residual; CFI= Comparative Fit Index.

**Table 3:** Linear Regression with, respectively, the EHQ total, Problems, Knowledge, and Feelings scores as dependent variables.

Dependent Variable: EHQ Total Score					
Model	R	R^2^	R^2^ Adapted	R^2^ Modified	ß
EAT-26-D	0.579	0.336	0.330	0.336	0.579
EAT-26-D; IPAQ-M	0.599	0.359	0.347	0.023	0.564; 0.152
Dependent variable: EHQ Problem score					
Model	R	R^2^	R^2^ adapted	R^2^ modified	ß
EAT-26-D	0.551	0.304	0.298	0.304	0.551
EAT-26-D; EAT-26-B&FP	0.616	0.380	0.369	0.076	0.371; 0.329
EAT-26-D; EAT-26-B&FP; IPAQ-M	0.661	0.437	0.422	0.057	0.331; 0.356; 0.241
Dependent variable: EHQ Knowledge score					
Model	R	R^2^	R^2^ adapted	R^2^ modified	ß
EAT-26-D	0.407	0.165	0.158	0.165	0.407
EAT-26-D; RSES	0.451	0.203	0.189	0.038	0.412; 0.195
EAT-26-D; RSES; EAT-26-OC	0.486	0.236	0.216	0.033	0.492; 0.204; −0.198
Dependent variable: EHQ Feelings score					
Model	R	R^2^	R^2^ adapted	R^2^ modified	ß
EAT-26-D	0.411	0.169	0.162	0.169	0.411

Note*:* EAT-26-D = EAT-Dieting subscale; EAT-26-B&FP = EAT- Bulimia and Food Preoccupation; EAT-26-OC = EAT-26-Oral Control; IPAQ-M = IPAQ-Moderate physical activity; RSES = RSES total score.

**Table 4:** Assessment of high versus low level of ON with disordered eating attitudes, self-esteem, and physical activity comparison among young adults: the significant results.

	Low EHQ (N = 67)	High EHQ (N = 68)	F _(1133)_	*p*	Partial η2
	M (SD)				
EAT-26_total	4.22 (6.86)	16.68 (12.19)	53.30	<0.001	0.29
EAT-26_Dieting	2.54 (5.16)	10.93 (7.58)	56.32	<0.001	0.30
EAT-26_Bulimia and food preoccupation	0.28 (0.62)	2.60 (3.27)	32.46	<0.001	0.20
EAT-26_Oral Control	1.09 (2.16)	2.84 (2.98)	15.24	<0.001	0.10
IPAQ_total	1625.34 (1419.73)	2919.85 (3024.81)	10.08	<0.01	0.07
IPAQ_Vigouros	434.03 (759.24)	1127.06 (1392.99)	12.83	<0.001	0.09

Note: EHQ = Eating Habits Questionnaire; EAT-26 = Eating Attitudes Test-26; RSES = Rosenberg Self-Esteem Scale; IPAQ = International Physical Activity Questionnaire; M = Mean; SD = Standard Deviation

## Data Availability

The dataset used during the current study are available from the corresponding author on reasonable request.
